# Supplementary material for: Assembly of Microparticles to Patterned Trenches Using the Depletion Volume Effect
Source: Micromachines (Basel). 2019 Jun 28;10(7):428. doi: 10.3390/mi10070428 (PMC6680554; doi:10.3390/mi10070428)
Supplement: Supplementary file 1 [file micromachines-10-00428-s001.pdf]

Assembly of microparticles to patterned trenches using the depletion volume effect

Yaoki Mori, Ryota Kawai, Hiroaki Suzuki*
Chuo University, Japan
Email: suzuki@mech.chuo-u.ac.jp

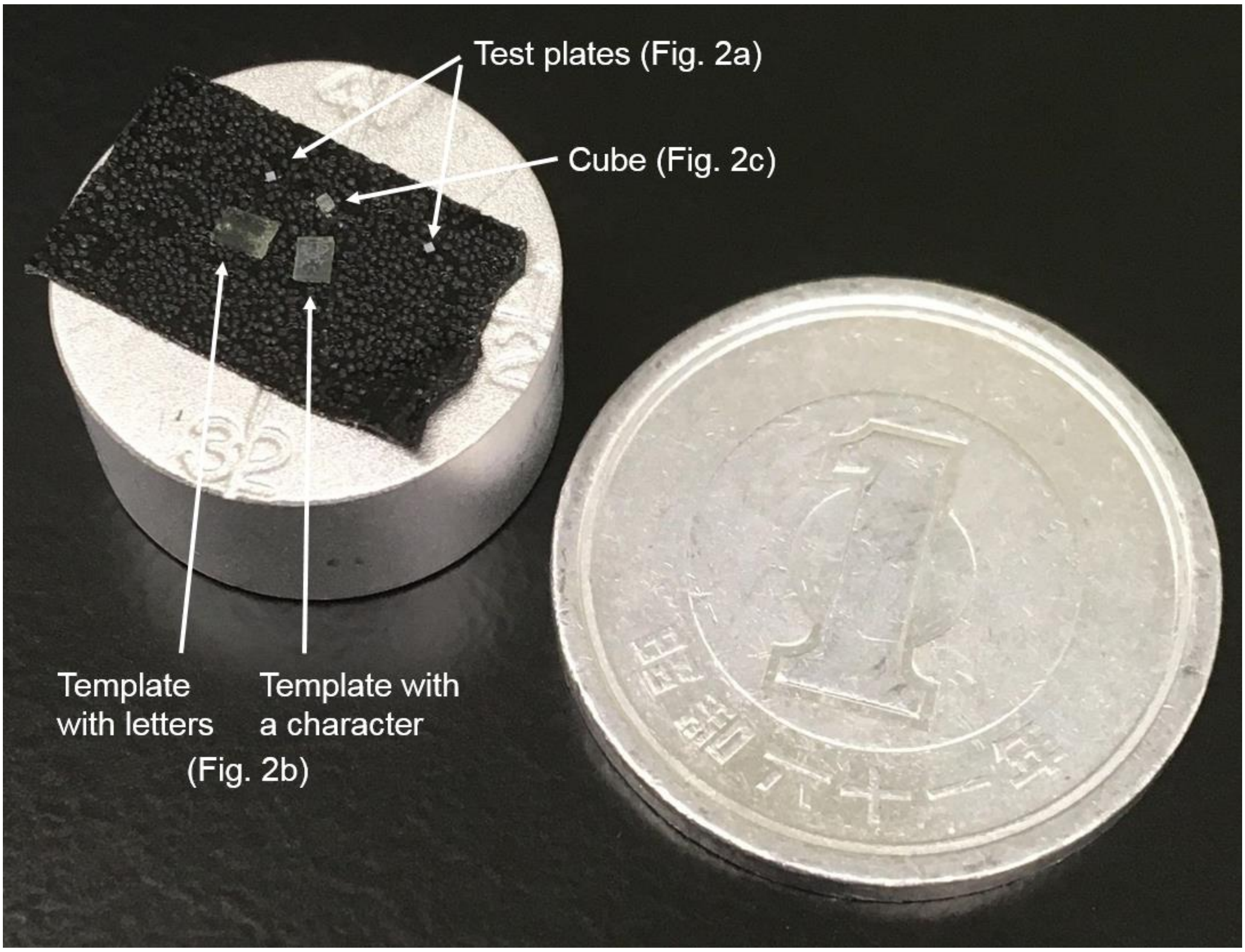

Figure S1 Comparison of the size of microfabricated templates to a 1-yen coin (20 mm diam.).

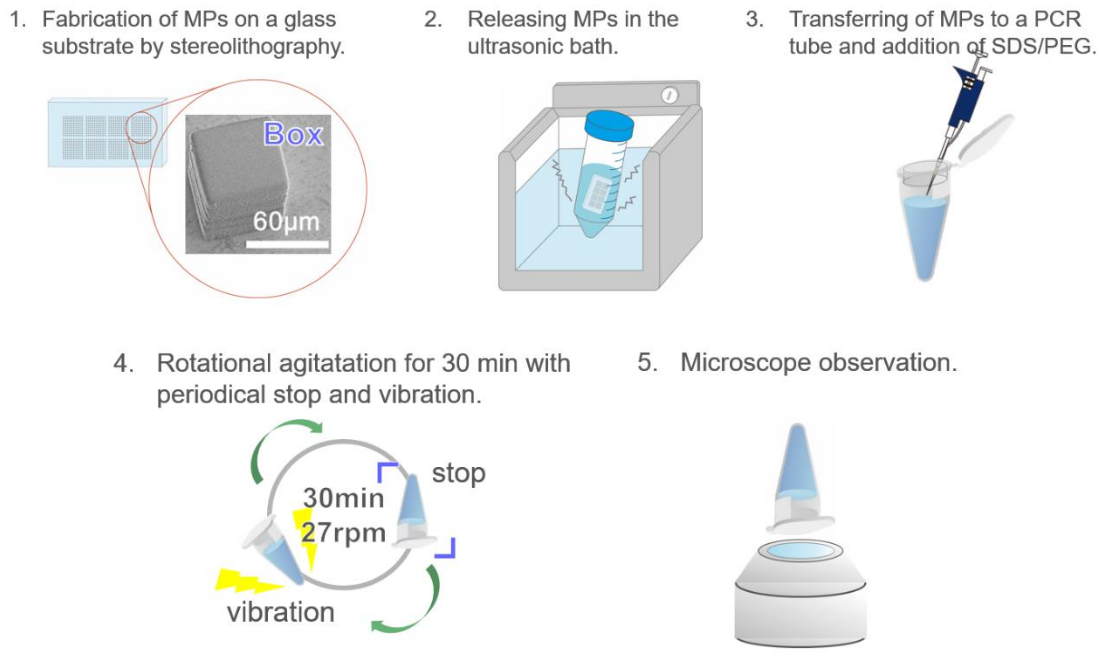

Figure S2 Schematic illustration of the experimental procedure.

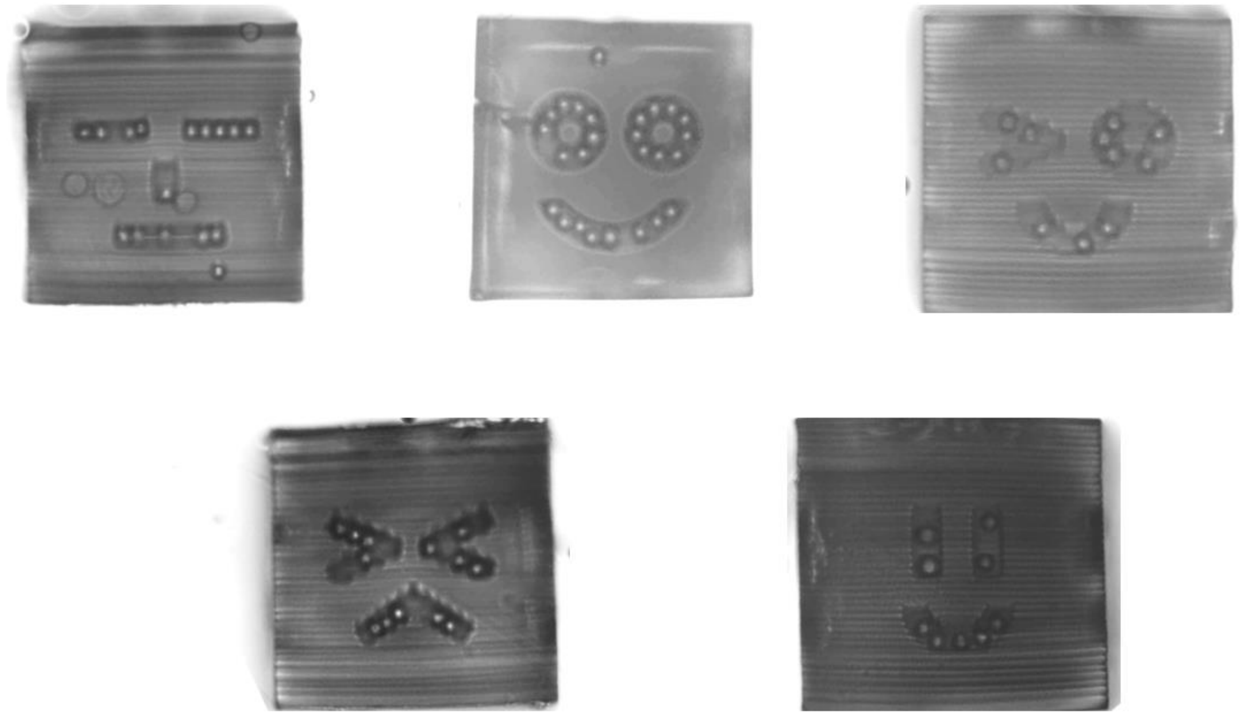

Figure S3 Assembly of beads to the cubic template with five faces obtained after 1.5h agitation.
